# A Comparison Between Sequential Conventional and Hypofractionated Boost Following Whole-Breast Radiotherapy: A Propensity Score-Matched Analysis

**DOI:** 10.7759/cureus.46913

**Published:** 2023-10-12

**Authors:** Jewel Rajan, Rajeev KR, Preethi Sara George, Asha Arjunan, Priya Balakrishnan, Paul Augustine, Beela Sarah Mathew

**Affiliations:** 1 Department of Radiation Oncology, Regional Cancer Centre, Thiruvananthapuram, IND; 2 Department of Epidemiology and Biostatistics, Regional Cancer Centre, Thiruvananthapuram, IND; 3 Department of Surgical Oncology, Regional Cancer Centre, Thiruvananthapuram, IND

**Keywords:** acute skin toxicity, boost dose, boost rt, hypofractionation, breast cancer

## Abstract

Background: The majority of local relapses after breast conservation therapy occur in the proximity of the primary lesion. Studies have shown that boost radiotherapy (RT) following conventional whole-breast radiotherapy (WBRT) of 50 Gy in five weeks improves outcomes. Boost RT also increases the risk of moderate skin reactions and fibrosis. The ideal boost RT dose and timing (sequential versus simultaneous) after hypofractionated radiotherapy schedules remain unclear. This retrospective propensity score-matched analysis assessed the outcome of sequential hypofractionated boost compared to conventional fractionated boost.

Methods: The study was approved by the Institutional Review Board of the Regional Cancer Centre, Thiruvananthapuram, India. Patients with stage I-III breast cancer who have received adjuvant radiotherapy with a sequential boost of either hypofractionated RT (8 Gy in three fractions) or conventional fractionated RT (10 Gy in five fractions) after conservative breast surgery were identified from the radiotherapy planning records and included in this study. A 1:1 case matching was performed using a propensity score incorporating four known prognostic factors, namely, clinical and pathological composite stage, tumor grade, tumor biology (based on estrogen and/or progesterone and HER2 neu expression), and boost technique, which may have an impact on acute toxicity to make the two boost groups more homogenous.

Results: After propensity score matching (PSM), there were a total of 166 patients, with 83 patients each in both conventional and hypofractionated boost RT groups. The median follow-up period was 30.7 months. At two years, locoregional recurrence-free survival (LRFS) was 98.8% in both groups. Disease-free survival (DFS) at two years for the hypofractionated group and conventional group was 91.5% and 96.3% (hazard ratio (HR): 2.5, 95% confidence interval (CI): 0.664-9.4, p = 0.161), respectively, with no statistically significant difference. Patients with grade 3 tumors who received hypofractionated boost had a statistically significant increased risk of recurrence (DFS: 88.9% versus 100%, HR: 60.559, 95% CI: 0.138-26613.2, p = 0.011). The overall survival (OS) at two years was 100% in both groups. There was no difference in acute skin toxicity between the two groups.

Conclusion: The present interim analysis shows similar locoregional recurrence-free survival, overall survival, and disease-free survival and acute skin toxicity for hypofractionated boost RT of 8 Gy in three fractions compared to the conventional boost of 10 Gy in five fractions. Hypofractionated boost is a feasible alternative option following hypofractionated whole-breast radiotherapy for women with breast conservation treatment. However, longer follow-up is required before forming definite conclusions.

## Introduction

Radiotherapy (RT) following breast conservation surgery (BCS) halves the risk of disease recurrence and reduces breast cancer mortality by about a sixth [1]. The risk of ipsilateral breast tumor recurrence (IBTR) must be reduced as much as possible while achieving good cosmetic and functional results. It has been demonstrated that the majority of local relapses occur in close proximity to the original lesion [2]. A number of randomized trials have shown that delivering a higher dose of radiation to the tumor bed (boost RT) has been associated with improved results [3-6]. However, there are concerns regarding both acute and late toxicities while increasing the total dose. Most of the boost RT trials delivered sequential 10-16 Gy in eight fractions following conventional whole-breast radiotherapy (WBRT) of 50 Gy in 25 fractions. The Lyon trial tested 10 Gy at 2.5 Gy per fraction (biological effective dose (BED) of 18.3 Gy and equivalent dose in 2 Gy fractions (EQD2) of 11 Gy) as tumor bed boost after conventional WBRT [4]. There were no significant differences in locoregional relapse and survival, but skin telangiectasia was more common in patients who received boost.

Hypofractionated WBRT is the current standard of care for patients undergoing BCS based on the Canadian and UK Standardisation of Breast Radiotherapy (START) trials [7-9]. Nevertheless, questions regarding the need for boost, the optimal dose, and the timing of the boost after hypofractionated WBRT remain unanswered. A phase II Korean study delivered WBRT of 39 Gy in 13 fractions followed by a boost of 9 Gy in three fractions (BED of 18 Gy and EQD2 of 10.8 Gy) [5]. The five-year locoregional recurrence was 1.4% at a median follow-up period of 57 months, and there was no grade 3 skin toxicity. At this hospital, the boost dose of 10 Gy in five fractions, which was routinely used following conventionally fractionated WBRT, was continued when hypofractionated WBRT was adopted in clinical practice. As efficacy and safety data regarding hypofractionation accumulated a sequential boost of 8 Gy in three fractions (BED of 15 Gy and EQD2 of 9 Gy), assuming α/β of 3 was used following WBRT of 40 Gy in 15 fractions (total BED of 80.72 Gy and EQD2 of 54.43 Gy).

This retrospective study is aimed at comparing the outcomes of patients who received a boost of either 10 Gy in five fractions (2 Gy/fraction) or 8 Gy in three fractions (2.67 Gy/fraction) following hypofractionated WBRT. An interim analysis at two-year follow-up is reported here.

## Materials and methods

This retrospective study is a propensity score-matched analysis of patients with stage I-III breast cancer, who underwent breast conservation surgery (BCS) followed by WBRT and tumor bed boost at the Regional Cancer Centre, Thiruvananthapuram, India, from January 1, 2019, to December 31, 2019. All patients who received WBRT followed by a sequential boost of either 10 Gy in five fractions or 8 Gy in three fractions were identified from the radiotherapy planning records. For those patients who received neoadjuvant chemotherapy prior to starting chemotherapy, skin tattoos were made around tumor margins by an oncology surgeon. Post chemotherapy, during wide excision of the lump, surgical clips were placed around the tumor bed to find the area for boost radiotherapy. Demographic details, tumor characteristics, treatment, acute toxicity, and follow-up information were collected from the patient records and radiotherapy prescriptions. Data regarding radiotherapy were captured from the radiotherapy planning system. The study was approved by the Institutional Review Board of the Regional Cancer Centre, Thiruvananthapuram, Kerala, India (01/2021/01).

Statistical analysis

Propensity score matching (PSM) is a statistical tool used to minimize the difference between baseline characteristics of two cohorts and make them more homogenous. When random allocation of participant interventions has not been carried out, propensity scores are an alternate technique to quantify the impact of an intervention. Intervention and control units with similar propensity score values, as well as possibly additional factors, are paired, and all unmatched units are discarded from the analysis.

Nearest-neighbor matching without replacement within a caliper was used. The caliper width was 0.2 standard deviations (SD) of the logit-transformed propensity score. A 1:1 case matching was performed using a propensity score incorporating four known prognostic factors, namely, clinical and pathological composite stage, tumor grade, tumor biology (based on estrogen and/or progesterone and HER2 neu expression), and boost technique, which may have an impact on acute toxicity.

The primary endpoint was locoregional recurrence, which was defined as recurrence in the treated breast and/or ipsilateral regional (axillary, internal mammary, and supraclavicular) lymph nodes. The secondary endpoints were disease-free survival (DFS), overall survival (OS), and acute skin toxicity. Disease-free survival was defined as the date from registration to the date of any breast cancer-related event such as locoregional recurrence, distant relapse, contralateral breast cancer, or death. Overall survival was calculated from the date of registration to the date of death from any cause or to the date of last follow-up.

Continuous variables are expressed as mean and standard deviation and categorical variables as counts and percentages. The chi-square test and Fisher's exact test were used for the comparison of categorical variables. Survival curves were obtained using the Kaplan-Meier method and were compared using the log-rank test. All p-values were two-sided, and values less than 0.05 were considered statistically significant.

## Results

From January 1, 2019, to December 31, 2019, 332 women had undergone BCS followed by WBRT and sequential boost RT. After propensity matching for specified covariates, a total of 166 patients were included in this analysis, with 83 patients in each group (8 Gy in three fractions (hypofractionated group) or 10 Gy in five fractions (conventional group)). The median follow-up period was 30.7 months (range: 2-46 months).

Patient, disease, and treatment characteristics

The mean age was 51 years and 53 years in the hypofractionated and conventional groups, respectively. Most patients were postmenopausal. Most of the patients in both groups had clinical stage II disease (50 in the hypofractionated group and 53 in the conventional group). The majority of the patients had hormone receptor (HR)-positive and HER2 neu-negative disease (49 in the hypofractionated group and 41 in the conventional group). Patient and disease characteristics prior to and following PSM are shown in Table 1.

**Table 1:** Patient and disease characteristics before and after PSM Values are presented as numbers (%). Abbreviations: PSM, propensity score matching; HR, hormone receptor; TNBC, triple-negative breast cancer; SD, standard deviation

Variables		Before PSM			After PSM		
		10 Gy/5# (n = 87)	8 Gy/3# (n = 245)	p-value	10 Gy/5# (n = 83)	8 Gy/3# (n = 83)	p-value
Age (years)	Mean (SD)	48.6 (10.6)	52.3 (10.5)	0.029	53 (10.5)	51.4(9.6)	0.312
	Range	22-81	26-79		25-76	26-79	
Menstrual status	Premenopausal	31 (35.6)	97 (39.6)	0.514	29 (34.9)	37 (44.6)	0.133
	Postmenopausal	56 (64.4)	148 (60.4)		54 (65.1)	46 (55.4)	
Family history	No	77 (88.5)	217 (88.6)	0.987	74 (89.2)	75 (90.4)	0.500
	Yes	10 (11.5)	28 (11.4)		9 (10.8)	8 (9.6)	
Laterality	Left	43 (49.4)	108 (44.1)	0.390	42 (50.6)	37 (44.6)	0.267
	Right	44 (50.6)	137 (55.9)		41 (49.4)	46 (55.4)	
Clinical stage	I	22 (25.3)	56 (22.9)	0.808	22 (26.5)	19 (22.9)	0.317
	II	56 (64.4)	167 (68.2)		53 (63.9)	50 (60.2)	
	III	9 (10.3)	22 (9)		8 (9.6)	14 (16.9)	
Pathology	Invasive ductal carcinoma	86 (98.9)	235 (95.9)	0.560	83 (100)	81 (97.6)	0.605
	Mucinous carcinoma	1 (1.1)	2 (0.8)		0 (0)	1 (1.2)	
	Invasive lobular carcinoma	0 (0)	8 (3.3)		0 (0)	1 (1.2)	
Grade	1	0 (0)	8 (3.3)	0.206	0 (0)	0 (0)	0.550
	2	30 (34.5)	92 (37.6)		30 (36.1)	27 (32.5)	
	3	57 (65.5)	141 (57.6)		53 (63.9)	55 (66.3)	
	Mucinous	0 (0)	4 (1.6)		0 (0)	1 (1.2)	
Tumor biology	HR positive, HER2 negative	41 (47.1)	155 (63.3)	0.001	41 (49.4)	49 (59.1)	0.416
	HR positive, HER2 positive	13 (14.9)	45 (18.4)		13 (15.7)	10 (12)	
	HR negative, HER2 positive	12 (13.9)	10 (4)		8 (9.6)	10 (12)	
	TNBC	21 (24.1)	35 (14.3)		21 (25.3)	14 (16.9)	

The whole-breast radiation dose was 40 Gy in 15 fractions for 165 patients, and one patient received 50 Gy in 25 fractions. Whole-breast radiotherapy was delivered using a three-dimensional conformal technique with tangent photon fields for all patients. In the hypofractionated group, 79 patients received photon boost and four received electrons. In the conventional boost group, 81 received photon boost and two received electron boost. The treatment characteristics are shown in Table 2.

**Table 2:** Treatment characteristics after PSM Values are presented as numbers (%). Abbreviations: PSM, propensity score matching; NACT, neoadjuvant chemotherapy; RT, radiotherapy

Treatment characteristics		Boost dose	
		8 Gy/3# (n = 83)	10 Gy/5# (n = 83)
Timing of surgery	Primary	65 (78.3)	69 (83.1)
	Post-NACT	18 (21.7)	14 (16.9)
Whole-breast RT dose	40 Gy/15#	83 (100)	82 (98.8)
	50 Gy/25#	0 (0)	1 (1.2)
Boost technique	Photon	79 (95.2)	81 (97.6)
	Electron	4 (4.8)	2 (2.4)
Adjuvant chemotherapy	Yes	53 (63.9)	60 (72.3)
	No	30 (36.1)	23 (27.7)
Adjuvant endocrine treatment	Yes	58 (69.9)	55 (66.3)
	No	25 (30.1)	28 (33.7)
HER2 positive (n = 41)	Yes	19 (22.9)	21 (25.3)
	No	1 (1.2)	0 (0)

One hundred thirty-four patients underwent primary surgery followed by adjuvant treatment, while 32 received neoadjuvant systemic treatment (NAST) and underwent surgery thereafter. The most commonly used chemotherapy regimen was a combination of anthracyclines and taxanes in both neoadjuvant and adjuvant settings. Ten out of 18 patients in the hypofractionated group and five out of 14 patients in the conventional group achieved a pathological complete response. Among the 41 patients with HER2-positive disease (20 in the hypofractionated group and 21 in the conventional boost group), all except one patient in the hypofractionated group received anti-HER2 therapy. All of the 113 patients with hormone receptor-positive disease (58 in the hypofractionated group and 55 in the conventional group) received adjuvant endocrine therapy.

Outcomes

At a median follow-up of 30.7 months, 11 patients relapsed, of which nine patients had distant metastases only, one had simultaneous distant and local recurrence, and one patient had local recurrence alone. The relapse pattern between the groups before and after PSM is summarized in Table 3. No patients died.

**Table 3:** Patterns of failure before and after PSM Values are presented as numbers (%). Abbreviation: PSM, propensity score matching

Variables	Before PSM		After PSM	
	10 Gy/5# (n = 87)	8 Gy/3# (n = 245)	10 Gy/5# (n = 83)	8 Gy/3# (n = 83)
Local relapse	1 (1.1)	4 (1.6)	1 (1.2)	1 (1.2)
Distant metastasis	3 (3.4)	17 (6.9)	3 (3.6)	7 (8.4)
Death	0 (0)	1 (0.4)	0 (0)	0 (0)

Locoregional recurrence

**Figure 1:**
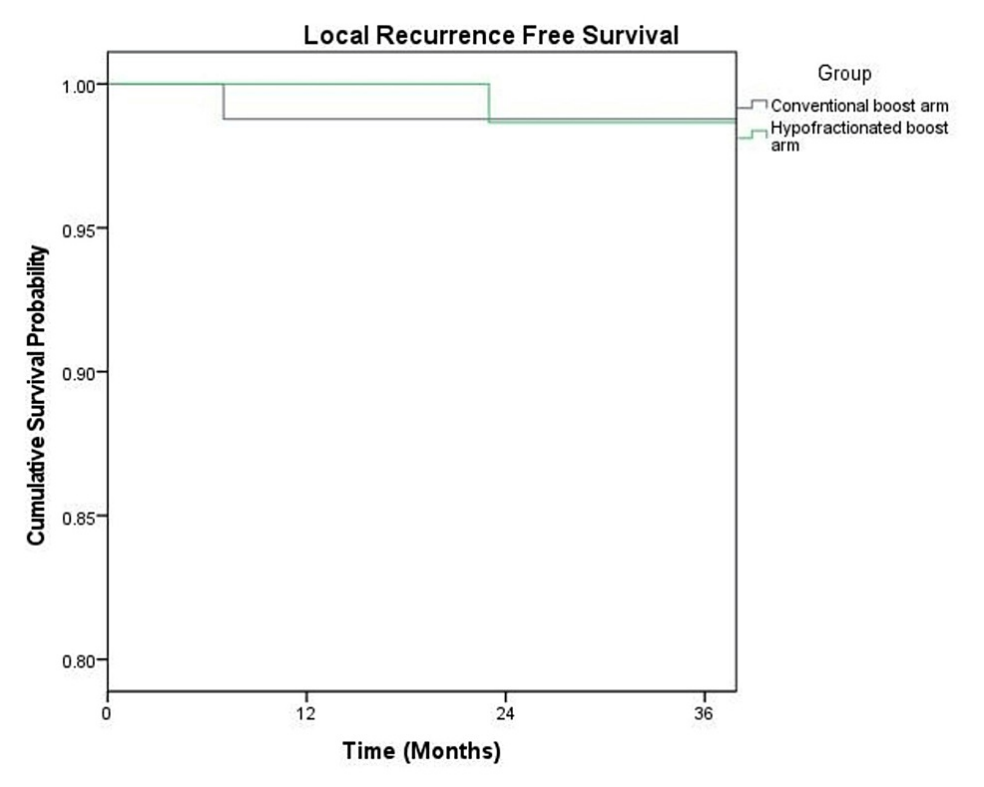
Kaplan-Meier curve showing LRFS Abbreviation: LRFS, locoregional recurrence-free survival

There were only two local relapses, one each in both arms, and both had triple-negative cancers. Isolated local recurrence was noted in the patient belonging to the conventional group. At two years, the locoregional recurrence-free survival (LRFS) was 98.8% in both groups (Figure 1).

Survival

Among the 11 patients with breast cancer-related events, eight belonged to the hypofractionated group and three to the conventional group. The DFS at two years for the hypofractionated group and conventional group was 91.5% and 96.3% (hazard ratio (HR): 2.5, 95% confidence interval (CI): 0.664-9.4, p = 0.161), respectively, with no statistically significant difference. The overall survival at two years was 100% in both groups.

**Figure 2:**
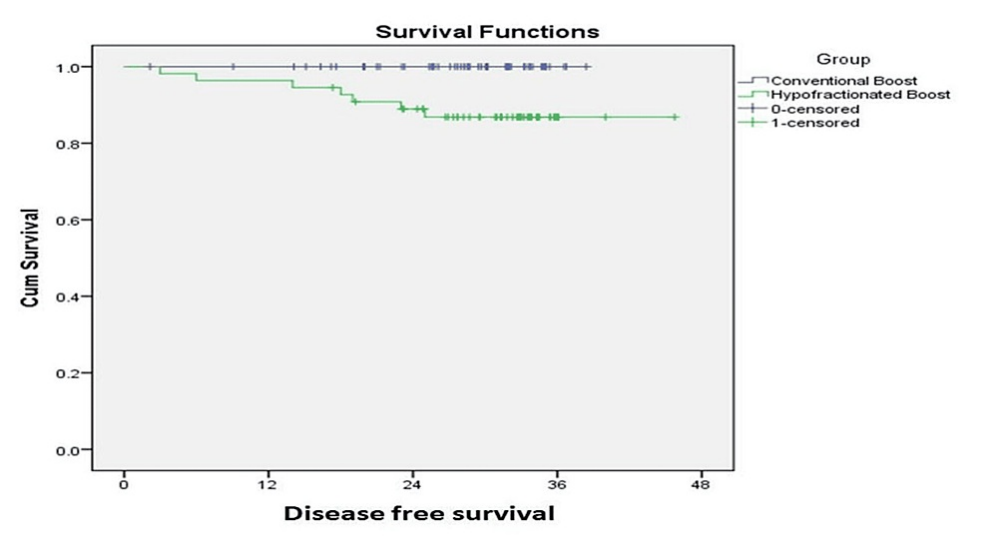
Kaplan-Meier curve showing DFS for grade 3 tumors Abbreviation: DFS, disease-free survival

Patients with grade 3 tumors who received hypofractionated boost had a statistically significant increased risk of recurrence (DFS: 88.9% versus 100%, HR: 60.559, 95% CI: 0.138-26613.2, p = 0.011) (Figure 2). Among the eight patients who relapsed in the hypofractionated group, seven had grade 3 tumors, while one patient had grade 2 cancer. There were no significant differences between the two groups in terms of DFS according to clinical and pathological stage, tumor biology, or boost delivery techniques (photons/electrons).

Acute skin toxicity

Acute skin toxicity (as per the Radiation Therapy Oncology Group (RTOG)/European Organization for Research and Treatment of Cancer (EORTC) morbidity scoring criteria) [10] is captured on a weekly basis during radiotherapy and at completion as a routine practice, and this information was available for all patients. The majority of the patients (n = 146, 88%) had grade 1 skin reactions only. Ten patients each had grade 2 and 3 skin reactions. Among the 10 patients who had grade 3 reactions, six patients were in the hypofractionated group, and four were in the conventional group (Table 4). There was no statistical significance between the groups (p = 0.567) for acute skin toxicity. Of the six patients who had grade 3 reactions in the hypofractionated group, five patients received photon boost and one received electron boost. Of the four in the conventional group, two each were treated with photon and electron boost. No statistically significant difference was shown according to the boost technique. One patient in the hypofractionated boost group developed grade 3 toxicity after 14 fractions (while on WBRT), and RT could not be continued due to a delay in healing. She did not receive the boost as planned.

**Table 4:** Acute skin toxicity and boost technique Values are presented as numbers (%).

Boost technique	Boost dose	Skin toxicity grade				
		Grade 1	Grade 2	Grade 3	Grade 4	p-value
Photon	8 Gy/3#	69 (83.1)	5 (6)	5 (6)	0 (0)	0.49
	10 Gy/5#	74 (89.2)	5 (6)	2 (2.4)	0 (0)	
Electron	8 Gy/3#	3 (3.6)	0 (0)	1 (1.2)	0 (0)	0.15
	10 Gy/5#	0 (0)	0 (0)	2 (2.4)	0 (0)	

## Discussion

The role of conventionally fractionated WBRT and boost has been demonstrated clearly by the EORTC trial [2]. At 10 years, the cumulative incidence of local recurrence was 6.2% for the boost group (4% improvement) with no impact on survival. This difference persisted for 20 years [3].

In our hospital, tumor bed boost is routinely practiced for all patients, except those of age 65 years or older with low-risk cancer. The boost dose used was 10 Gy in five fractions, except for those with postoperative margins with focal positivity of in situ carcinoma, where 16 Gy in eight fractions is delivered as boost. Hypofractionated WBRT became a standard of care based on long-term results from pivotal trials conducted in the UK and Canada [7-9]. With a significant shortening of overall treatment time, this resulted in improved patient satisfaction. As efficacy and safety data regarding hypofractionation for WBRT as well as boost accumulated, a sequential boost of 8 Gy in three fractions was used following WBRT of 40 Gy in 15 fractions (total BED of 80.72 Gy and EQD2 of 54.43 for α/β of 3 Gy). In this interim analysis with propensity score matching, only two local failures (one was associated with distant relapse) were reported at a median follow-up of 30.7 months. The LRFS at two years was 98.8% for both hypofractionated and conventional boost groups. Short-term results indicate that the hypofractionated boost dose of 8 Gy in three fractions yielded similar outcomes compared to 10 Gy in five fractions. The Lyon boost trial, which tested a sequential boost dose of 10 Gy in four fractions daily, reported a 3.6% local relapse rate at 10 years [4]. A phase II study from Korea tested WBRT 39 Gy in 13 fractions with a sequential boost of 9 Gy in three fractions [5]. With a median follow-up of 57 months, the five-year locoregional recurrence was 1.4%, and the disease-free survival rate was 97.4%. Both the Lyon trial and the Korean study delivered a BED of 18 Gy and EQD2 of 11 Gy to the tumor bed as boost. A similar PSM study by Lee et al. including 178 patients compared with hypofractionated WBRT with boost and conventional WBRT with boost reported no difference in IBTR [11]. The American Society for Radiation Oncology (ASTRO) guidelines published in 2018 also permitted the use of hypofractionated boost [12].

There was no statistically significant difference in either DFS or OS between the two treatment groups in this study. There was no death in either group, and the overall survival was 100%. This is similar to outcomes published by prior studies on the impact of boost and those employing hypofractionated boost [3-5]. In the EORTC study, the 20-year distant recurrence was 24.8% in the no-boost group against 26% in the boost group (p = 0.29). The overall survival was 59.7% and 61.1% (p = 0.323) in the boost group and no-boost group, respectively. A Cochrane series of data from 2,810 events in three studies with 6,549 women found no difference in disease-free survival with or without the addition of boost (HR: 0.94, 95% CI: 0.87-1.02, p = 0.12) [13]. The study by Lee et al. also showed no difference in DSF and OS between patients receiving hypofractionated or conventional doses [11].

A statistically significant decline in DFS was noted for grade 3 cancers treated with 8 Gy in three fractions in this analysis (88.9% versus 100%, p = 0.011). Over two-thirds of the patients included in this analysis had grade 3 cancers. This suggests that 8 Gy in three fractions may be an inferior dose for such patients. No such difference has been noted in other studies. No statistically significant difference in terms of DFS was noted with regard to clinical and pathological stage, tumor biology, or boost delivery techniques (photons/electrons) between the two groups.

Skin toxicity and adverse cosmesis are of concern with regard to tumor bed boost. In the present analysis, 146 patients had only grade 1 acute skin toxicity. There was no significant difference between the groups with regard to acute skin toxicity. Only six patients received electron boost. No difference in acute skin toxicity could be shown according to the boost technique. In the study by Romestaing et al., grade 1 and 2 toxicities were reported in 12.4% of patients who received boost compared to 5.9% of those who did not [4]. This retrospective study could not obtain data on cosmesis.

One of the main advantages of hypofractionated schedules is the shortening of overall treatment time. This not only helps patients but is also particularly important in hospitals with large patient loads and radiotherapy waiting times. In recent years, hypofractionated radiotherapy incorporating simultaneous boost (SIB) has been reported to have equal efficacy and safety and shortens the treatment period further [14,15]. However, the optimal technique for SIB delivery is not yet defined and has implications with regard to labor-intensive radiotherapy planning and more stringent quality assurance and may not be easily accepted into wide clinical practice. Sequential hypofractionated boost may be a more feasible approach in such centers.

The main limitations of this study are its retrospective design, small number of patients, and short follow-up. The event numbers were small, and hence, no definite conclusions can be drawn. Long-term morbidity data needs to be collected. Other factors linked to toxicities include breast size and boost volume [16]. These could not be assessed in this study.

## Conclusions

The short-term analysis of a sequential hypofractionated boost of 8 Gy in three fractions following hypofractionated WBRT appears to be a feasible option with similar outcomes in terms of local control, disease-free and overall survival, and acute skin toxicity compared to a boost of 10 Gy in five fractions at least in patients with low-grade cancers. Nevertheless, larger studies and longer follow-ups are required before forming definite conclusions.
